# Influence of Lactation Stage on Content of Neurotrophic Factors, Leptin, and Insulin in Human Milk

**DOI:** 10.3390/molecules29204973

**Published:** 2024-10-21

**Authors:** Elena Sinkiewicz-Darol, Katarzyna Łubiech, Iwona Adamczyk

**Affiliations:** 1 Department of Physiology and Toxicology, Faculty of Biological Sciences, Kazimierz Wielki University, Chodkiewicza 30 St., 85-064 Bydgoszcz, Poland; katarzyna.lubiech@ukw.edu.pl (K.Ł.); iwona.adamczyk@ukw.edu.pl (I.A.); 2 Human Milk Bank, Ludwik Rydygier Provincial Polyclinical Hospital in Torun, St. Josef 53-59 St., 87-100 Torun, Poland

**Keywords:** human milk, leptin, insulin, neurotrophic factors, nutritional programming, lactation stages

## Abstract

Human milk comprehensively meets the nutritional needs of a child, providing not only structural and energy components but also various bioactive factors. Among these, neurotrophic factors and hormones involved in metabolic processes deserve special attention. Studies using enzyme-linked immunosorbent assays compared the content of neurotrophic factors—CNTF, NT-3, and NGF—and hormones, leptin and insulin, in two groups of breast milk samples: early lactation (1–3 months) and extended lactation (>6 months, up to 12 months). The results indicated changes in leptin and insulin levels as the lactation period extended. NGF, leptin, and insulin were present in milk samples from both study groups, with leptin and insulin levels being higher in the early lactation group. CNTF and NT-3 were not detected in any of the samples from either study group. The analyses confirmed that human milk from women who breastfeed for extended periods remains a source of biologically active components and macronutrients that support a child’s development and health.

## 1. Introduction

Newborns are exposed to many factors that may negatively affect their health [1,2]. Among other elements, breast milk provides protection during this earliest period of life, as it is rich in ingredients that not only ensure the proper growth and development of the child but also provide substances with bioactive effects, including those that have a direct impact on nutritional programming [3,4,5]. Human milk is considered the most suitable food for newborns and infants. The potential benefits of breastfeeding have been linked to the presence of various growth and neurotrophic factors in breast milk. Many studies indicate better results of neurodevelopmental tests for breastfed children compared to formula-fed children [6,7,8]. Due to its proven short- and long-term health benefits, breastfeeding is recognized by the World Health Organization (WHO), the American Academy of Pediatrics (AAP), and the European Society for Paediatric Gastroenterology, Hepatology and Nutrition (ESPGHAN) as the gold standard in newborn and infant nutrition [9]. Additionally, in the case of sick and prematurely born children, who, due to idiopathic or other health-related reasons, cannot be fed their own mother’s milk in accordance with WHO and AAP recommendations, the next choice after their mother’s milk should be donor milk from human milk banks [10]. Despite intensive research on the composition of breast milk, little is known about its components and their impact on the developing child during extended lactation (beyond 6 months). The stages of lactation are among the most important determinants of the composition of individual components in milk [11,12]. Research has shown that the content of macronutrients and bioactive components in human milk adapts to the developmental needs of the child as lactation progresses [13,14].

Adipokines are one of the most extensively studied groups of biological compounds, the dysregulation of which may contribute to the development of overweight and obesity [15,16,17]. Numerous studies have highlighted the crucial role of neurotrophic factors in the mechanisms underlying the development of metabolic diseases [18,19,20].

The development of structures essential for the functioning of the nervous system, such as the cerebellum, occurs particularly intensively during the final stages of pregnancy and the early postnatal period. The regulation of axonal and dendritic branching is especially important, as it is vital for the proper functioning of neuronal circuits. For this reason, a premature birth carries a high risk of structural abnormalities in the central nervous system [21]. Early childhood is also a period of rapid synaptogenesis, which is crucial for proper bodily development [22]. Neurotrophic factors that influence the differentiation and maturation of neurons, as well as those involved in synaptogenesis, are essential for the healthy development of neural structures. Human milk, as a source of postnatal neuroprotective factors, can therefore play a significant role by providing these biologically active components. Since breast milk remains the sole source of nutrients until a child reaches six months of age, in accordance with WHO recommendations, it becomes especially important during this critical developmental phase. The proper development of brain structures, such as the cerebellum, is important for long-term health and is associated with a reduced risk of neurodegenerative diseases, which are a major concern in modern societies [23]. The components of human milk that significantly impact an infant’s development include neurotrophic factors such as neurotrophin 3 (NT-3), ciliary neurotrophic factor (CNTF), nerve growth factor (NGF), leptin, and insulin [24,25,26,27].

CNTF, NT-3, and NGF are neurotrophic factors that regulate the survival, differentiation, and maturation of new neurons; the growth of axons; and the formation of synapses during prenatal, neonatal, and adult life [24]. CNTF is a pluripotent neurotrophic factor that was originally isolated from chick embryonic ciliary neurons. It affects the development and proper functioning of the nervous system, as well as adipocytes, muscle cells, cardiomyocytes, osteoblasts, and immune cells [28]. Ciliary neurotrophic factor is essential for proper body weight management [18]. Neurotrophin 3 and nerve growth factor belong to the family of neurotrophic factors, also known as neurotrophins. Both are essential for the differentiation and survival of specific neuronal subpopulations in the central and peripheral nervous systems [29,30]. The neurotrophin family comprises at least four proteins: NGF, BDNF (brain-derived neurotrophic factor), NT-3, and NT-4/5. These cytokines are synthesized as prepropeptides and are proteolytically processed to produce mature proteins [31]. NT-3 is expressed in brown and beige adipocytes and regulates sympathetic nervous system innervation and energy metabolism [20]. Nerve growth factor is involved in the inflammatory context of obesity and metabolic syndrome [32]. CNTF, NT-3, and NGF have been identified in breast milk in the early stages of lactation [33,34,35]. So far, there are no clear data on the content of these factors in milk beyond the sixth month of lactation. An extremely important component of breast milk is leptin, which has a multidirectional action. Primarily, it regulates the feelings of satiety and hunger, interacting with neurotrophic factors, among others. Along with other adipokines, it influences the nutritional programming of newborns and infants. Leptin secretion is regulated by neurotrophic factors [36,37].

Insulin, a hormone secreted primarily by the β-cells of the islets of Langerhans in the pancreas, plays an important role in glucose homeostasis, cell growth, and metabolism. Insulin is primarily responsible for regulating the metabolism of all macronutrients—carbohydrates, proteins, and fats—although it does not directly participate in all these processes [38]. Currently, little is known about the presence of leptin and insulin in breast milk during extended lactation or whether they can influence the eating behavior of a breastfed child at this stage of development.

Given the increasing percentage of infants and young children at risk of overweight and obesity later in life, resulting from factors such as the feeding model in infancy—and WHO recommendations on the duration of breastfeeding and its preventive effects on lifestyle diseases—it is extremely important to understand the composition of human milk and, in later stages, the mechanisms through which its individual components influence the developing child. The aim of the study was to analyze the content and correlations of bioactive components in human milk—nerve growth factor, ciliary neurotrophic factor, neurotrophin 3, leptin, and insulin—as well as macronutrients, in samples collected from women assigned to two groups: (1) Group 1—women in the first months of lactation (1–3 months; N = 30), EL (early lactation); and (2) Group 2—women over 6 months of lactation (up to 12 months; N = 30), LTL (long-term lactation).

## 2. Results

### 2.1. Content of Leptin, Insulin, and Neurotrophic Factors in Human Milk Samples

The results of enzyme immunoassay analyses showed the presence of both leptin and insulin in all analyzed samples. In the case of NGF, its content was detected in 47% (N = 14) of the samples from the EL group and 53% (N = 16) of the samples from the LTL group.

The concentrations of leptin, insulin, and NGF in the milk samples from the two analyzed groups differed, with leptin ranging from 46 to 723 pg/mL and from 20 to 408 pg/mL, and insulin ranging from 4 to 64 uIU/mL and from 6 to 31 uIU/mL in the EL and LTL groups, respectively. NGF ranged from 28 to 363 pg/mL in the milk samples from early lactation and from 18 to 349 pg/mL in the milk samples from extended lactation. The leptin, insulin, and NGF contents in the milk samples from the analyzed groups are shown in Table 1. No measurable CNTF or NT-3 was detected in any of the samples tested.

**Figure 1:**
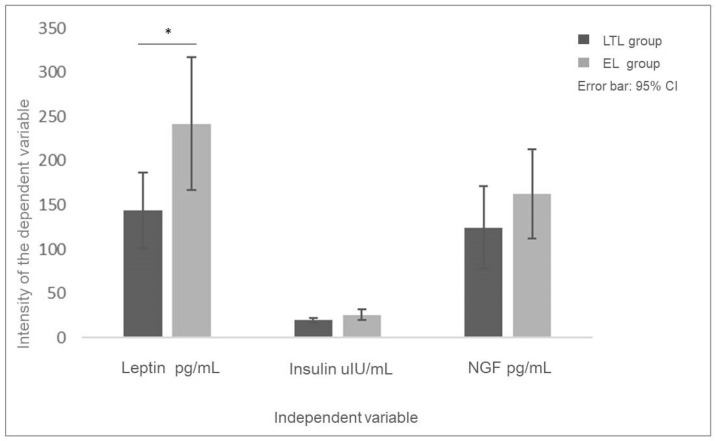
The comparison of leptin, insulin, and NGF content in milk samples from the LTL (long-term lactation, >6 months up to 12 months) and EL (early lactation, 1–3 months) groups. * statistically significant.

A statistically significant difference was observed between the groups in terms of leptin levels (*p* = 0.032). Based on the mean values, the leptin level was found to be higher in the milk samples from the early lactation group compared to those from the long-term lactation group [Table 1], with the difference between the groups being moderately large (Cohen’s *d* = 0.62) [Figure 1]. The analysis showed a statistically insignificant difference in insulin levels (*p* = 0.063). Analyzing the mean insulin levels in both groups, it was found that the insulin content in the milk samples was slightly higher in the EL group compared to the LTL group, and the effect size index indicated a moderately strong difference between the groups (Cohen’s *d* = 0.53) [Table 1, Figure 1].

The analysis did not reveal statistically significant differences between the groups in terms of NGF levels. Additionally, no relationship was identified between the presence of nerve growth factor in the milk samples and the levels of the other tested factors.

### 2.2. Content of Macronutrients in Human Milk Samples

**Figure 2:**
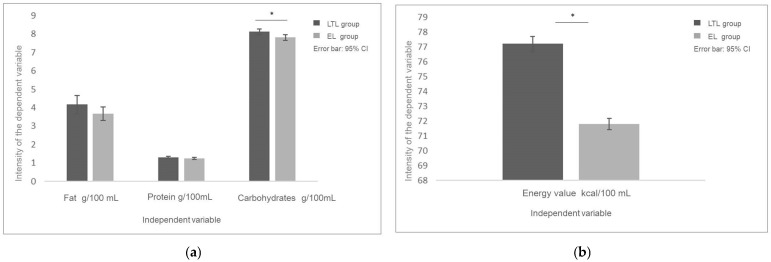
The content of macronutrients in the analyzed milk samples. (**a**) The comparison of the content of fat, total protein, and carbohydrates in milk samples from the long-term lactation (>6 months of lactation) and early lactation groups (1–3 months of lactation); (**b**) the comparison of the energy value of milk samples from the long-term lactation and early lactation groups. * statistically significant.

The analysis showed statistically significant differences between the groups in terms of carbohydrate levels (*p* = 0.005). Based on the mean values, it was found that the level of carbohydrates in the milk samples from the long-term lactation group was higher compared to those from the early lactation group (8.12 ± 0.38 g/mL vs. 7.81 ± 0.38 g/mL), and the difference between the groups was large (Cohen’s *d* = 0.81) [Figure 2a]. A statistically significant difference in the energy value of the milk was also identified, although the *p* value was marginal (*p* = 0.080). The results for this variable indicate a higher energy value of the milk from the LTL group compared to the EL group (77.20 ± 11.87 kcal/mL vs. 71.81 ± 9.61 kcal/mL) [Figure 2b]. The effect size was moderate (Cohen’s *d* = 0.50).

### 2.3. Analysis of the Relationship Between the Content of Insulin, Leptin, NGF, and Macronutrients in Human Milk Samples

In the LTL group, the analysis showed a statistically significant, positive, and moderately strong relationship between the leptin and insulin contents in the milk samples (r = 0.47, *p* = 0.012). Thus, as the level of one of these components in the milk increased, the content of the other component also increased. The relationship between the fat content in the milk samples and their energy value was also found to be statistically significant (r = 0.99, *p* < 0.001). The higher the fat content recorded in the milk samples, the higher their energy value [Table 2].

In the early lactation group, a very strong positive correlation between the fat content in the milk and its energy value was observed (r = 0.96, *p* < 0.001). A positive and moderately strong relationship between the leptin and insulin levels was observed; however, it was not statistically significant.

Additionally, statistically significant, positive, and strong relationships between the carbohydrate content and the fat content (r = 0.51, *p* = 0.008) and energy value (r = 0.57, *p* = 0.002) of the milk were noted. This indicates that as the carbohydrate content in the milk samples increased, the fat content and energy value of the milk in the early lactation group also increased. A negative and moderately strong relationship between the insulin level and the total protein content in the milk was observed, which was significant at the level of statistical tendency (r = −0.39, *p* = 0.052). Higher insulin levels were associated with lower total protein content. Significant, positive, and moderately strong relationships were also found between the NGF level and both the carbohydrate content and energy value of the milk, significant at the level of statistical tendency. Higher NGF levels corresponded with higher carbohydrate content and higher energy values in the milk samples from the EL group [Table 3].

## 3. Discussion

Studies on animal models have shown that neurotrophins and their receptors are present in various organs, such as the oviduct, uterine segments, and mammary gland, during pregnancy and lactation [30,39]. The exact mechanism of neurotrophic factor transfer into human milk has not been identified. Many studies highlight the significant role of CNTF and NT-3 in fetal development, suggesting that they also play a critical role in postnatal development. Research on the content of these two factors in breast milk is limited. Only a few studies have reported the presence of CNTF and NT-3 in human milk.

In a study by Collado et al., NT-3 and CNTF were identified in both colostrum and mature milk samples obtained from mothers of premature (N = 20) and full-term (N = 20) infants. It has been shown that the stage of lactation affects the content of these factors in milk. The expression of NT-3 and CNTF was significantly higher in mature pre-term milk compared to mature-term milk samples [40]. In a study of milk samples in the early stages of lactation (N = 14, including colostrum samples), CNTF was identified at levels of 10–50 ng/mL, showing individual variability, but the percentage of samples in which CNTF was detected was not specified. The study was conducted on a small group, with only 14 milk samples [33]. Milk samples from this study that contained CNTF were used for culture experiments. Further experimental studies showed that breast milk containing CNTF, as well as GDNF, promoted neurite growth.

In our study, NT-3 and CNTF were not detected in any of the analyzed samples. The main reason may be the critical role these factors play in central nervous system development during fetal life. Therefore, we did not observe these factors in mature and long-term lactation milk samples from healthy women who gave birth to healthy, full-term infants. To verify this hypothesis, it is necessary to conduct tests on milk samples from women who gave birth to extremely and late premature babies. The content of neurotrophic factors in milk may also be influenced by other factors, such as the health status of the mother (e.g., pregnancy-induced hypertension, preeclampsia, gestational diabetes mellitus) and the infant. None of the participants in our study had any chronic diseases. The influence of geographical factors on the composition of milk cannot be ruled out. The BDNF neurotrophic factor has been identified in several studies on human milk in various populations, including the Polish population [41,42,43]. In a recent cross-sectional study by Perrin et al. (2019) conducted on 74 milk samples, without restrictions regarding the lactation period and using the same research methodology as other authors, the presence of BDNF was not detected in any of the samples tested [44].

Nerve growth factor is another neurotrophic factor receiving increased attention for its presence in human milk and its potential impact on the developing child. NGF is important for neuronal plasticity and the survival of cholinergic neurons, which are associated with memory. Additionally, NGF plays a key role in memory and cognitive functions [45]. Recent studies have reported that NGF also plays a key role in angiogenesis [29]. In our study, the mean NGF level was higher in milk samples from the early stages of lactation (162.83 ± 93.07 pg/mL) compared to those from prolonged lactation (124 ± 92.56 pg/mL). The NGF levels in the tested samples were characterized by high individual variability. The observed differences were not statistically significant. This suggests that NGF may be necessary for the proper development of the newborn, not only immediately after birth but also during later stages of infancy. This hypothesis is supported by studies from other researchers, which show that in colostrum (3 days after delivery), the mean NGF level was 283.0 ± 208.3 pg/mL, almost twice as high as in mature milk [46]. Colostrum is the first milk produced after birth, characterized by a well-documented composition and significant clinical importance for both full-term and premature newborns. In terms of bioactive components, its composition differs significantly from that of transitional or mature milk, sometimes containing 2–3 times higher concentrations of certain compounds or compounds not present at later stages of lactation [47].

Our results are consistent with previously published studies, which reported that NGF levels in mature milk remain within the range of 300–400 pg/mL [46]. Research by Dangat et al. (2014) also observed that NGF concentrations increased as lactation progressed. It is important to note that this study was conducted on a different ethnic group and was a follow-up analysis that highlighted individual variability, specifically in women with preeclampsia [35]. Additionally, it has been shown that subclinical and clinical mastitis, as well as prior COVID-19 infection, reduce NGF levels in human milk [42]. In the early lactation group, a higher carbohydrate content and energy value were correlated with elevated NGF levels. However, this trend was not observed in the long-term lactation group. To date, no studies have examined the correlation between human milk macronutrients and NGF content. Studies conducted on premature infants have shown that NGF levels in blood serum are influenced by protein and energy intake. Recent research indicates that premature infants have lower plasma NGF levels compared to full-term neonates [48]. It is assumed that neurotrophic factors, like other components of human milk, may support normal infant development during the postnatal period. Some studies have shown significant clinical correlations between the level of circulating NGF in the newborn and neurodevelopmental outcomes [30]. Our study shows that NGF is identified not only in mature milk, but also in prolonged lactation.

Recent studies also suggest the involvement of nerve growth factor in nutritional programming, where macronutrients play a key role [49,50]. Neurotrophins and mast cells have been shown to have a metabotrophic effect and are involved in the metabolism of carbohydrates and lipids. NGF, like other neurotrophic factors, including the well-documented BDNF, regulates leptin levels. Leptin and NGF have been shown to regulate adipose tissue function. In our studies, we did not observe any relationship between NGF content and the levels of leptin and insulin in the tested milk samples [49,50].

The source of adipokines in breast milk is not clearly defined. Some of them, including leptin, are synthesized in the mammary gland. Adipokines, including leptin, are peptide hormones released mainly by adipocytes. They play a significant role in regulating metabolic functions in various tissues, including the adipose tissue, liver, brain, and muscle. The role of leptin in child development has been well documented. This hormone plays a role in maintaining energy homeostasis, influencing the infant’s metabolism and body weight, and regulating appetite and food intake [34]. Research conducted on three groups of infants (small for gestational age, large for gestational age, and appropriate for gestational age) suggests that the leptin in breast milk may influence the child’s growth and appetite, as well as regulate nutritional processes at an early stage of life [51]. Hormones in breast milk, such as leptin, adiponectin, and ghrelin, may influence long-term appetite signaling and have a preventive effect on the development of obesity [52]. Leptin also participates in the development of the central nervous system structures [53,54]. Leptin is particularly important during the third trimester of pregnancy, when large amounts of this hormone pass from the placenta to the fetal body. A significant issue in neurological development is the deficiency of this factor in children born prematurely, which is related to limited endogenous production and the cessation of intrauterine supplies of the hormone. Therefore, breast milk becomes a valuable source of leptin after delivery [55]. This is confirmed by studies that show higher levels of leptin in the serum of breastfed infants than in formula-fed infants [56,57]. Leptin also affects other processes in the body, including regulating blood pressure, hematopoiesis, and angiogenesis, and performs immunoregulatory functions that help alleviate intestinal inflammation or stimulate immune system cells, e.g., T lymphocytes [58]. The presence of leptin in breast milk at subsequent stages of lactation suggests that its biological role does not end at the early stage of breastfeeding and is also biologically important in the growth and development of older breastfed children during prolonged lactation. Further studies are needed to determine the effect of human milk adipokines from long-term lactation on a child’s development.

Bronsky et al. (2011) conducted research on breast milk samples from 72 healthy women over 12 months of lactation. The leptin content after the first, third, sixth, and twelfth months of lactation was 0.3 ± 0.04, 0.20 ± 0.03, 0.1 ± 0.01, 0.1 ± 0.02, and 0.2 ± 0.04 ng/mL, respectively [59]. An initial decrease in the leptin content in breast milk samples was observed along with a subsequent increase in the case of prolonged lactation (approximately 12 months), which the authors explained by extending the child’s diet to include solid products and, consequently, extending the intervals between feedings. Also, research conducted by Brunner et al. (2015) indicated the presence of leptin in various periods of lactation and a tendency for its content to decrease over time. Breast milk samples were collected at 6 weeks (N = 152) and 4 months (N = 120) after delivery [60]. Research conducted by Schuster et al. (2011) was based on analyzing breast milk samples from the first half-year of lactation [53]. In the first month of lactation, the leptin levels remained stable (0.17 ng/mL). In the next month of lactation, the leptin concentration was significantly lower (0.11 ng/mL). The hormone concentrations then increased slightly and remained at a stabilized level until the 6th month of lactation (0.15 ng/mL) [50]. Ilcol et al. (2006) were also interested in analyzing the leptin content at various stages of lactation. In their study, 160 samples of breast milk from various stages of lactation were examined: the first 3 days (N = 37; colostrum), 4–14 days (N = 27; transitional milk), 15–30 days (N = 16; early mature milk), 31–90 days (N = 37; mature milk), and 91–180 days (N = 43; late mature milk). In these studies, colostrum was characterized by the highest leptin concentration (3.28 ± 0.41 ng/mL). Later, the leptin level gradually decreased [61].

The results we obtained also indicated the presence of leptin in the composition of human milk despite the passage of time, although comparing the results obtained in the group of breast milk samples from the first three months of lactation and the group after six months, a significant decrease in its content can be observed (242.07 ± 196.72 and 143.48 ± 115.74 pg/mL, respectively). Based on the results obtained, it can be assumed that leptin may play a role in nutritional programming and neurological development, probably throughout the lactation period, although its role is most important during the first period of breastfeeding.

Studies in animal models have provided insights into the effects of insulin on developing organisms. Insulin can interact with the intestinal mucosa via receptors located on both the apical and basolateral surfaces of enterocytes. As a result, insulin plays a role in the maturation of the gastrointestinal tract, the secretion of mucosal enzymes, and the mechanisms regulating intestinal passage [62]. Research suggests that insulin may retain its biological activity after ingestion by infants and pass intact from the gastrointestinal tract into the bloodstream [63]. However, the impact of insulin in breast milk on the weight and growth of breastfed infants has not been fully elucidated. Contradictory findings suggest either a negative correlation between infant weight, BMI-for-age z-scores, and fat-free mass, or no effect at all [27,64].

Studies have shown that insulin levels in human milk correlate with those observed in blood serum. Such a relationship was also observed among breastfeeding women with type 1 diabetes, in whom insulin is exogenous in origin. These findings suggest that insulin is transported into breast milk regardless of whether its origin is endogenous or exogenous [60]. Shehadeh et al. (2003) observed that the concentration of insulin in breast milk decreased from day 3 to day 10, with values of 50.1 ± 34.6 and 41.1 ± 28.5 microU/mL, respectively. A significant decrease in insulin content in breast milk was noted in the breast milk samples from women who gave birth at term [65].

Other studies examining longer time intervals indicate that insulin concentrations are lowest in colostrum, followed by an increase in mature milk [64]. Similar observations were made by Yu et al. (2018), who analyzed hormone content, including insulin, in breast milk collected at 3, 42, and 90 days postpartum. An increase in insulin levels was observed between days 3 and 42 (20.41 μU/mL and 28.20 μU/mL, respectively). In the following interval, between 42 and 90 days postpartum, insulin concentrations decreased (from 28.20 μU/mL to 24.61 μU/mL) [66]. In our study, we observed a decrease in insulin concentration in the breast milk samples from the long-term lactation group compared to the early lactation group. It has been shown that there is a relationship between individual hormones, the macronutrient composition of breast milk, and the characteristics of breastfed children. A study by Ilcol et al. (2006) reported a correlation between leptin and insulin concentrations in mature milk (r = 0.331, *p* < 0.05) [61]. Our study also indicates a correlation between insulin and leptin concentrations (r = 0.36; *p* = 0.068). A negative correlation was observed between the total protein content of human milk and insulin concentration (r = −0.39, *p* = 0.052). In addition, a study by Mazzocchi et al. (2019) indicates an inverse correlation between the leptin and insulin content in breast milk and the trajectory of weight-for-length z-scores and BMI-for-age z-scores in infants at four months of age [67].

Macroelements in human milk, such as fats, carbohydrates (primarily lactose), and protein, are essential for the proper growth and development of newborns. During the early neonatal period, breast milk is often the sole source of nutrients, making the energy provided by human milk critical for infant growth. The development of metabolomics techniques has enabled the precise determination of the composition and dynamic changes occurring in human milk, as well as the metabolic pathways it influences. The macronutrient content of human milk exhibits significant variability, with its composition differing between mothers based on factors such as the gestational age, lactation stage, time of day, and feeding session [68]. The most substantial daily variations are observed between colostrum, transitional milk, and mature milk. Moreover, differences in the composition of human milk have been noted among mothers of pre-term infants. In pre-term milk, the content of macro- and micronutrients, as well as bioactive components, fluctuates and adapts to the infant’s immediate nutritional and developmental needs [69]. Research has demonstrated that not only protein content but also the ratio of protein to energy and carbohydrates to fat in breast milk may influence the growth rate of breastfed infants [70]. The appropriate supply of macronutrients is crucial at every stage of a child’s development.

Our previous research has shown that milk produced after one year of lactation remains a valuable source of macronutrients and does not lose its nutritional value. Additionally, it serves as a reservoir of bioactive compounds, suggesting that human milk adapts to meet the needs of the developing infant [12,13]. In this study, the macronutrient levels in breast milk beyond the sixth month of lactation remained constant. The observed changes included a higher carbohydrate content and increased energy value in milk samples from extended lactation compared to those from the initial stages of lactation. This may be related to the infant’s increased energy demand beyond six months of age.

An important area requiring further research is the bioavailability of the nutrients supplied through human milk to the infant. To date, research has primarily focused on differences in the digestion and bioavailability of active ingredients in early lactation for pre-term versus full-term infants [71,72]. In studies on the nutritional physiology of breastfed infants during the first year of life, Gridneva et al. (2018) demonstrated that the concentration and calculated daily intake of adipokines, such as leptin, influence infant body composition [73]. However, the exact mechanisms governing the bioavailability of leptin in breast milk have yet to be fully described. Further research on the supply and bioavailability of other components of human milk, including neurotrophic factors, is also necessary.

## 4. Materials and Methods

### 4.1. Study Participants

The study group consisted of 60 breastfeeding women who were recruited via the Regional Human Milk Bank at the Ludwik Rydygier Provincial Polyclinic Hospital in Torun, Poland, during the period from March 2023 to July 2023. All study participants were healthy, non-smoking women with normal BMI (18.5–24.9) and uncomplicated full-term pregnancies. All neonates and infants were in good health condition. The inclusion criteria for the study were as follows: age 18 or over; the lactation period for the control group of 1–3 months postpartum, and for the study group, >6 months postpartum (up to 12 months); term birth; feeding the child with one’s own milk (directly from the breast or expressed milk); and the possibility of obtaining a minimum of 40–50 mL of material for testing in the 24 h breast milk collection procedure developed for the study. Data concerning the study participants came from questionnaires completed at the time of recruitment and milk collection. The exclusion criteria for the study were as follows: obese women suffering from metabolic and chronic diseases, delivery before 37 weeks of pregnancy, multiple pregnancies, insufficient milk supply to meet their child’s nutritional needs, and the lack of the participant’s consent to participate in testing and to collect milk for testing.

### 4.2. Milk Sampling

All study participants received written instructions on how to collect and store material for the analysis. Milk samples were collected during the 24 h period in four time intervals: from 6:00 to 12:00, from 12:00 to 18:00, from 18:00 to 24:00, and from 24:00 to 06:00. Women expressed 10–15 mL of milk before and 10–15 mL after breastfeeding in each time interval. In the case of women who fed their babies with a teat bottle, 15 mL samples were taken from all the expressed milk. The HM samples collected during the 24 h period were stored under cooling conditions (4–8 °C) and then immediately transported in a cooling box to a laboratory. The recommended refrigeration time for a milk sample from the last feeding or pumping session is a maximum of 12 h before shipment to the laboratory. The milk samples from each mother were pooled separately for a further analysis. Breast milk for the analysis was transferred into sterile containers designed for storing human milk. The raw milk was immediately tested for macronutrient content.

### 4.3. Determination of Essential Macronutrients

The MIRIS Human Milk Analyzer (HMA, Miris AB, Uppsala, Sweden) was used to analyze macronutrients, fat (g/100 mL), total protein and crude protein (g/100 mL), total solids (g/100 mL), and energy content (kcal/100 mL), in milk samples. The MIRIS HMA is based on semi-solid mid-infrared (MIR) transmission spectroscopy. The wave ranges used in the device are specific to the following groups: carbonyl (5.7 µm) for fat, amide groups (6.5 µm) for protein, and hydroxyl groups (9.6 µm) for carbohydrates. A daily zero-setting check procedure was performed before the analysis using the calibration solution provided by the supplier. Total protein refers to the content based on the total amount of nitrogen in a sample; non-protein nitrogen compounds are also included in this value. True protein is the value corrected for non-protein nitrogen compounds and represents only the actual protein content. The MIRIS HMA uses a factor of 6.38 to convert nitrogen content into protein content. Before the analysis, each sample was heated to 40 °C in a thermostatic bath and then homogenized using the MIRIS Sonicator (Miris AB, Uppsala, Sweden) at 1.5 s per mL. Each sample was analyzed in duplicate, with the resulting value being the average of the two measurements.

### 4.4. Determination of Bioactive Components

All bioactive components were determined using the ELISA method. Before the initial analysis, milk samples were centrifuged at 12,000 rpm for 20 min at 4 °C. The aqueous phase of human milk was used for the assay of CNTF, NT-3, NGF, leptin, and insulin. The assay detecting particular components in the milk was performed at least twice. The concentrations of leptin, insulin, NGF, NT-3, and CNTF were analyzed with commercial ELISA kits using microplates pre-coated with a monoclonal antibody specifically for the test substances. The following tests were used: Human Leptin (R&D Systems, Inc., Minneapolis, MN, USA); Human CNTF (R&D Systems, Inc. Minneapolis, MN, USA); Insulin ELISA (DRG Instruments GmbH, Marburg, Germany); Human NT-3 (Novus Biologicals, Minneapolis, MN, USA); and Human NGF (Novus Biologicals, Minneapolis, MN, USA). For the study, the serum/plasma option was chosen as the most suitable for human milk. The method was pre-tested on various sample dilutions as proposed in the protocol. As a result of this validation, the final analyses were performed on undiluted milk samples. Each sample in the ELISA assay was measured in duplicate. The amounts of CNTF, NT-3, NGF, and leptin were expressed as pg/mL, and insulin as µIU/mL. The detection of leptin, insulin, NT-3, NGF, and CNTF was performed using a microtiter reader (SPECTROstar Nano, BMG LABOTECH, Ortenberg, Germany) set to 450 nm with the reference filter. For the data analysis, SPECTROstar Nano Data Analysis Software (Mars version) was used.

### 4.5. Statistical Analysis

Mean values and standard deviations of the mean were determined. Statistical analyses were performed using the IBM SPSS Statistics 29 package. It was used to analyze basic descriptive statistics, including the Shapiro–Wilk test, Student’s *t*-test for independent samples, and Pearson’s r correlation analysis. The significance level for this analysis was set at α = 0.05. In turn, *p* values between 0.05 and 0.1 were considered to indicate a statistical trend. Before the main analyses, descriptive statistics were calculated along with the Shapiro–Wilk normality test for quantitative variables. These analyses were performed separately for the two groups.

A total of six samples were excluded from the analyses: one due to missing data (N = 1) and five due to extreme outliers (N = 5) in the remaining data. Ultimately, the calculations were carried out on a group of 54 women: N = 26 in Group 1 (EL, early lactation) and N = 28 in Group 2 (LTL, long-term lactation).

## 5. Conclusions

In conclusion, our results show that human milk during 6 months of lactation can be an important source of bioactive components for nutritional programming, such as leptin, insulin, and neurotrophic factors. Within macronutrients, the variability concerns mainly the carbohydrate content and energy value, which is related to the child’s greater energy requirement during the second half of the first year of life.

## Figures and Tables

**Table 1:** Comparison of Leptin, Insulin, and NGF Content in Milk Samples Between Analyzed Groups.

	Long-Term Lactation Group (N = 28)		Early Lactation Group (N = 26)					95% CI		
Dependent Variable	M	SD	M	SD	*t*	*df*	*p*	LL	UL	Cohen’s *d*
Leptin, pg/mL	143.48	115.74	242.07	196.72	−2.22	39.84	0.032 *	−188.23	−8.95	0.62
Insulin, uIU/mL	19.57	7.61	25.82	14.89	−1.92	36.61	0.063	−12.84	0.36	0.53
NGF, pg/mL	124.60	92.56	162.83	93.07	−1.09	26	0.287	−110.51	34.05	0.41

N—number of observations; M—mean; SD—standard deviation; *t*—value of the test statistic; *df*—degrees of freedom; *p*—statistical significance; CI—confidence interval for the difference between means; LL and UL—lower and upper limits of the confidence interval; * statistically significant.

**Table 2:** The Relationship Between the Content of Insulin, Leptin, and NGF Levels and the Content of Macronutrients in Milk Samples from the Long-Term Lactation Group: Results of Pearson’s r Correlation Analysis.

Parameter		Leptin, pg/mL	Insulin, uIU/mL	NGF, pg/mL	Fat, g/100 mL	Total Protein, g/100 mL	Carbohydrates, g/100 mL	Energy Value, g/100 mL
Leptin, pg/mL	*r* Pearson’s		0.47	0.05	0.02	−0.03	−0.29	−0.04
	*p* value		0.012 *	0.867	0.917	0.872	0.158	0.855
Insulin, uIU/mL	*r* Pearson’s	0.47		−0.21	0.19	0.00	0.23	0.20
	*p* value	0.012 *		0.443	0.362	0.996	0.269	0.345
NGF, pg/mL	*r* Pearson’s	0.05	−0.21		−0.18	−0.19	−0.17	−0.21
	*p* value	0.867	0.443		0.530	0.493	0.551	0.460
Fat, g/100 mL	*r* Pearson’s	0.02	0.19	−0.18		−0.25	0.00	0.99
	*p* value	0.917	0.362	0.530		0.226	0.987	<0.001 *
Total protein, g/100 mL	*r* Pearson’s	−0.03	0.00	−0.19	−0.25		−0.15	−0.22
	*p* value	0.872	0.996	0.493	0.226		0.475	0.288
Carbohydrates, g/100 mL	*r* Pearson’s	−0.29	0.23	−0.17	0.00	−0.15		0.11
	*p* value	0.158	0.269	0.551	0.987	0.475		0.590
Energy value, g/100 mL	*r* Pearson’s	−0.04	0.20	−0.21	0.99	−0.22	0.11	
	*p* value	0.855	0.345	0.460	<0.001 *	0.288	0.590	

NGF, nerve growth factor; * statistically significant correlations are marked in bold.

**Table 3:** The Relationship Between the Content of Insulin, Leptin, and NGF Levels and the Content of Macronutrients in Milk Samples from the Early Lactation Group—Results of Pearson’s r Correlation Analysis.

Parameter		Leptin, pg/mL	Insulin, uIU/mL	NGF, pg/mL	Fat, g/100 mL	Total Protein, g/100 mL	Carbohydrates, g/100 mL	Energy Value, g/100 mL
Leptin, pg/mL	*r* Pearson’s		0.36	0.27	0.26	−0.13	0.22	0.26
	*p* value		0.068	0.375	0.202	0.538	0.284	0.194
Insulin, uIU/mL	*r* Pearson’s	0.36		0.17	−0.10	−0.39	−0.04	−0.07
	*p* value	0.068		0.584	0.646	0.052	0.856	0.723
NGF, pg/mL	*r* Pearson’s	0.27	0.17		0.42	−0.01	0.48	0.48
	*p* value	0.375	0.584		0.149	0.977	0.094	0.093
Fat, g/100 mL	*r* Pearson’s	0.26	−0.10	0.42		−0.05	0.51	0.96
	*p* value	0.202	0.646	0.149		0.825	0.008 *	<0.001 *
Total protein, g/100 mL	*r* Pearson’s	−0.13	−0.39	−0.01	−0.05		0.21	0.03
	*p* value	0.538	0.052	0.977	0.825		0.306	0.896
Carbohydrates, g/100 mL	*r* Pearson’s	0.22	−0.04	0.48	0.51	0.21		0.57
	*p* value	0.284	0.856	0.094	0.008 *	0.306		0.002 *
Energy value, g/100 mL	*r* Pearson’s	0.26	−0.07	0.48	0.96	0.03	0.57	
	*p* value	0.194	0.723	0.093	<0.001 *	0.896	0.002 *	

NGF: nerve growth factor; * statistically significant correlations are marked in bold.

## Data Availability

The data supporting the findings of this study are available upon request from the corresponding author.
